# The Mediator Roles of Problematic Internet Use and Perceived Stress Between Health Behaviors and Work-Life Balance Among Internet Users in Germany and China: Web-Based Cross-Sectional Study

**DOI:** 10.2196/16468

**Published:** 2020-05-11

**Authors:** Lingling Gao, Yiqun Gan, Amanda Whittal, Song Yan, Sonia Lippke

**Affiliations:** 1 Department of Psychology & Methods Jacobs University Bremen Bremen Germany; 2 School of Psychological Cognitive Sciences and Beijing Key Laboratory of Behavior and Mental Health Peking University Beijing China

**Keywords:** healthy lifestyle, work-life balance, internet, healthy diet, exercise, culture

## Abstract

**Background:**

Work-life balance is associated with health behaviors. In the face of digitalization, understanding this link requires a theory-based investigation of problematic internet use and perceived stress, which are so far unknown.

**Objective:**

On the basis of the compensatory carry-over action model, this study aimed to determine whether problematic internet use and perceived stress mediate the relationship between health behaviors and work-life balance in two groups of internet users from different environments (residents in Germany and China). We also investigated whether the place of residence was a moderator.

**Methods:**

An online questionnaire (N=877) was administered to residents from Germany (n=374) and China (n=503) in 3 languages (German, English, and Chinese). Moderated mediation analyses were run with health behaviors as the independent variable, work-life balance as the dependent variable, problematic internet use and perceived stress as the mediator variables, and place of residence as a potential moderator.

**Results:**

On a mean level, individuals in Germany reported less problematic internet use and more health behaviors than individuals in China; however, they also had lower work-life balance and higher perceived stress. Results showed that health behaviors seem to be directly related to work-life balance in both groups. Among the residents of Germany, a partial mediation was revealed (β=.13; *P*=.01), whereas among the residents of China, a full mediation was found (β=.02; *P*=.61). The mediator role of perceived stress was compared with problematic internet use in all the serial models and the parallel model. Residence moderated the relationship between health behaviors and work-life balance: The interrelation between health behaviors and work-life balance was stronger in Germany (β=.19; *P*<.001) than in China (β=.11; *P*=.01) when controlling for other variables.

**Conclusions:**

The findings of this study are in line with the compensatory carry-over action model. To promote work-life balance, individuals should perform health behaviors to help overcome problematic internet use and perceived stress. Both problematic internet use and perceived stress mediated health behaviors and work-life balance partially in German study participants and fully in Chinese study participants.

## Introduction

### Work-Life Balance

Increasingly, many people struggle with maintaining a work-life balance [1] and have to manage issues arising from a poor work-life balance, such as sickness, limitations in the ability to work, and absence of work [2]. There has been increasing attention on how best to achieve a work-life balance in the modern society, which has been investigated by researchers from different perspectives. Work time (eg, long work hours) has been found to have a negative association with work-life balance [3-5]. When exploring the ways to decrease this issue, it was found that the internet can help change the traditional work model to a more flexible model. The use of emails, smartphones, and home computers, for example, makes it possible to work without having a typical *nine-to-five* workday [6], which may help to maintain a work-life balance. However, it was also found that excessive internet use can decrease work-life balance [7-9]. Perceived stress was also found to be an important factor that affects work-life balance negatively [10], but which could be mitigated by health behaviors [9]. Previous evidence has indicated that health behaviors (eg, regular physical activity and healthy diet) help to prevent stress [11]. Moreover, health behaviors, such as regular physical activity and healthy nutrition of employees, have also been recommended to maintain a work-life balance [12-14].

### Health Behaviors

Common important health behaviors include both health-promoting behaviors [15], for instance, physical activity [16,17] and a healthy diet (eg, sufficient water intake and fruit and vegetable intake) [18,19], and health-risk behaviors [16,17], such as smoking status [16,20,21] and alcohol consumption status [22,23]. Previous studies have found that different single health behaviors are interrelated [16], such as diet and exercise [24,25] and smoking and alcohol consumption [18]. There is increasing focus on how to help employees maintain their work-life balance. Besides efforts by employers, employees can also help themselves to improve their work-life balance by performing health behaviors after work. Employees’ health behaviors are the focus of this study that sought to explore how people’s health behaviors interrelate with work-life balance.

### Perceived Stress and Problematic Internet Use as Mediators

As described earlier, not only health behaviors but also problematic internet use and perceived stress have been found to play a role in work-life balance [10,26]. The *compensatory carry-over action model* by Lippke [27] provides an approach to understanding the underlying psychological and behavioral mechanisms of multiple behaviors related to an outcome. However, the model has not yet been applied to the main focus of this paper, that is, work-life balance, internet use, and health behaviors. According to this model, different behaviors are not isolated; rather, they interact with each other in complex ways. Emotionally relevant higher-level goals (such as work-life balance) drive different health behaviors by initiating and strengthening behavior-specific intentions [27]. If individuals have set a higher-level goal and did not reach this goal (eg, because of situations such as work stress), their well-being is affected [28-30]. As previous studies have indicated, most people experience different stressors within their lives, which can interfere with adopting and maintaining behaviors [27]. In line with this model, perceived stress has been found to be a negative indicator of quality of life [31-33]. Moreover, it has been found that stress acts as a mediator between work-family conflict and psychological health [34]. Previous evidence has also indicated that health behaviors (eg, regular physical activity and healthy diet) help to prevent stress [11] and maintain a work-life balance [12-14]. Among a sample of working individuals, higher perceived stress was found to be associated with a higher fat diet and less physical activity [35,36]. Perceived stress may be one factor that undermines the positive relationship between health behaviors and work-life balance. However, the mediator role of perceived stress between health behaviors and work-life balance has received limited attention in previous research. This study is a novel effort to fill this gap, in particular, to help understand the interrelation of different health behaviors and internet use behavior on the basis of the *compensatory carry-over action model*.

Previous studies have also found that poor health behaviors, such as inadequate nutrition intake, were related to problematic internet use [7-9]. According to the *compensatory carry-over action model* [27], the outcome experience (eg, problematic internet use) from one behavior (eg, internet use behavior) may also affect a higher-level goal (eg, work-life balance) together with another behavior (eg, health behavior). Although the internet may help to arrange flexible work time and in turn maintain work-life balance [6], for some people, problematic internet use can also harbor hazards to their work-life balance [7-9]. Moreover, time is a finite resource, the use of which must be divided among individual needs, work, and family needs [37]. Problematic internet use may occupy the time one could spend on physical activity, family, and work and is thus negatively related to work-life balance. Excessive internet use has been found to be associated with behaviors that negatively affect people’s health, mood, work, and occupation [38]. So far, little is known about the relationship between health behaviors and work-life balance, while also considering the risk brought by problematic internet use. Owing to the large number of people suffering from the consequences of problematic internet use [39], it is important to examine how health behaviors combined with problematic internet use interrelate with work-life balance.

Some studies have indicated that problematic internet use has a direct impact on perceived stress [40,41], for example, it was found that problematic internet use is related to perceived stress and negative feelings, such as depression and anxiety [42,43]. Other studies have shown that perceived stress plays an important role in problematic internet use [44,45]. According to the *compensatory carry-over action model* [27], both perceived stress and problematic internet use may mediate the relationship between health behaviors and a higher-level goal (ie, work-life balance).

In this study, this underlying mechanism of this relationship was explored by comparing two possible serial models, with perceived stress and problematic internet use as serial mediators. If problematic internet use is assumed to influence perceived stress, then the chain is as follows: health behavior → problematic internet use → perceived stress → work-life balance. If one assumes that perceived stress influences problematic internet use, then the chain is as follows: health behavior → perceived stress → problematic internet use → work-life balance.

### Place of Residence Differences Among Study Variables

Previous studies have shown large cultural differences between China and Germany [46,47]. For example, according to Hofstede’s empirical framework, Germany is representative of a *Western* country and individualistic society, in which people consider their needs over the needs of the organization to which they belong. Conversely, China is representative of an *Eastern* country and collectivistic society, in which people put the needs of the organization before individual needs [48-52].

On the basis of these differences, previous studies comparing work-life balance between *Western* and *Eastern* companies revealed that work-life balance ranks higher in Western companies than in Eastern companies, as more attention is given to it [53]. The contexts of work-life conflict and solution in China are significantly different from those found in Western countries [54]. Some studies have found that in Eastern countries, family, including elderly parents, may provide advice and emotional support when people need help in maintaining a work-life balance [55,56]. Culture has been found to moderate the relationship between work resources and work-family conflict [57]. Although health behaviors as personal resources [58] were found to be associated with work-life balance [12-14], the moderator role of the place of residence between health behaviors and work-life balance still needs to be investigated.

The difference in health behaviors between Western countries, such as European countries and the United States, and Eastern countries, such as China, was investigated in previous studies [59,60]. Factors such as the geographic, historical, and economic development stage of a country and the coping mechanisms of its citizens may affect people’s health behaviors, such as food preferences and physical activity [61,62]. For example, the number of facilities for physical activity provided by the government (eg, parks) may inhibit or facilitate participation in physical activity, depending on the socioeconomic status [62]. It has been found that fruit intake and physical activity have higher prevalence in Western countries [63], and the percentage of nonsmokers is higher in Eastern countries than that in Western counties [63]. Differences in perceived stress exist between Western and Eastern countries as well [64,65]. For problematic internet use, previous studies have shown that Eastern counties have a higher internet addiction score than Western countries [66,67].

### Proposed Research Questions

On the basis of these previous research findings and the *compensatory carry-over action model*, this study sought to understand and explore the mechanisms underlying the relationship between health behaviors and work-life balance.

The following two major research questions were addressed:

1. Do problematic internet use and perceived stress mediate the relationship between health behaviors and work-life balance in both Germany and China?
2. Does the country of residence moderate the relationship between a healthy lifestyle and work-life balance?

## Methods

### Participants

The online survey included 877 participants (538/877, 61.3% women). German (98/877, 11.2%) and English (276/877, 31.5%) versions of the questionnaire were used to collect data in Germany (group 1: residents in Germany), and a Chinese (503/877, 57.4%) version of the questionnaire was used to collect data in China (group 2: residents in China). A minimum sample size of 233 was used in both groups. The participants’ ages ranged from 17 to 65 years (mean 30.0, SD 10.8).

### Procedure

Data were gathered from residents in middle-sized cities in Germany (Bremen) and China (Shijiazhuang). As Germany is the second most popular migration destination in the world [68] and English is widely spoken in Germany, both German and English versions of the questionnaire were provided for the participants to freely choose the language they prefer (no significant differences were found between the German and English versions of the questionnaire in the pilot analyses). Residents in China were provided with the Chinese version of the questionnaire.

The online questionnaires were used to collect data from October 2016 to August 2018 through email (in Germany and China), Facebook (in Germany), and link distribution face to face (with the help of the research assistants in Germany and with the help of primary school teachers who distributed the questionnaires among the pupils’ parents at parent-teacher conferences in China). Participants answered multiple-choice questions by clicking the appropriate check box and open questions by inputting text content. On the first page of the survey, all participants were informed of the confidentiality, anonymity, and voluntary nature of their responses and given the opportunity to obtain the study results by providing contact information on the page. Those who clicked the box to provide their informed consent could continue to the questionnaire pages. The study received ethical approval by the Ethics Commission of the German Association of Psychology (Deutsche Gesellschaft für Psychologie, EK-A-SL022013). The link for the survey was sent to the staff and students in universities. Study participants were also asked to forward the invitation and the link to families and friends in Germany and China.

### Measures

*Health behavior* was operationalized from the summation of 5 items: physical activity, sufficient water consumption, nutrition, smoking status, and alcohol consumption status. For the purposes of analysis, each behavior was coded into a binary variable, with 0 representing the unhealthy option. *Physical activity* was assessed with the short form of the International Physical Activity Questionnaire [69], which has been tested for use in adults and has shown cross-cultural validity and reliability [70]. This questionnaire covers mild, moderate, and strenuous physical activities. Participants indicated the duration (in minutes) and frequency (in days) of each activity domain per week. Minutes per week were multiplied by days per week to obtain a sum score per activity domain and then added for total activity per week. Total physical activity was classified as 1 (those who fulfilled the World Health Organization’s recommendation of ≥2.5 hours per week) and 0 (physical activity <2.5 hours per week).

S*ufficient water consumption* was assessed by the question, “Please think about your typical weeks, do you drink 1.5 L of non-alcoholic and non-caffeinated beverage (water, juice, fruit and herbal tea) during the day?” and it was possible to answer with yes (1) or no (0). This question has been shown to have acceptable reliability and validity [16]. *Nutrition* was measured by the question, “Please think about your typical weeks, do you eat five or more servings of fruit and vegetable per day?” and was to be answered with yes or no. This question has also been shown to have acceptable reliability and validity [71]. *Smoking status* was assessed by asking participants “Are you a smoker?” Answers were classified into 1 (nonsmoker or ex-smoker) and 0 (occasional smoker or regular smoker). The *alcohol consumption status* was assessed by asking participants “Do you drink alcohol on a regular basis?” and was classified as 1 (no) and 0 (yes). Both the smoking and alcohol questions have been shown to have acceptable reliability and validity [72]. All different health behaviors were summed, with 0 indicating the fewest health behaviors performed and 5 indicating the performance of all health behaviors on the recommended level.

*Work-life balance* was measured by using a 5-item scale [73] consisting of employees’ satisfaction with their achieved balance between work and private life. Answers were on a 5-point Likert scale, ranging from 1 (strongly disagree) to 5 (strongly agree). Sample items were “I am satisfied with the balance between my work and private life.”; “It is difficult for me to balance my work and private life”; and “I am meeting the requirements of both my work and my private life.” The Cronbach alpha was .81 in this study.

*Perceived stress* was measured with 2 questions from the Perceived Stress Scale [74], as in previous studies [75]—“In the last month, how often have you felt nervous and stressed?” and “In the last month, how often have you felt difficulties were piling up so high that you could not overcome them?”—and it was rated on a 5-point Likert scale. The Cronbach alpha was .75 in this study.

Problematic internet use was assessed with the Internet Addiction Questionnaire (ISS-10r) [76]. The current version includes 10 items with answer scores on a scale of 1 to 4 ranging from completely disagree to agree completely (eg, “I often spend more time on the internet than I intended”). For the evaluation of problematic internet use, ISS-10r sum values of the 5 subscales (loss of control, withdrawal symptoms, development of tolerance, negative consequences on work and performance, and negative consequences on social relationships) were utilized. The total score can vary from 20 to 80 and distinguishes 3 types of internet use: no problematic internet use (20-49), problematic internet use tendency (50-59), problematic internet use (60-80). The ISS-10r is one of the validated tools to assess problematic internet use, and the Cronbach alpha was .81 in this study.

Participants also answered sociodemographic questions, including those on age, gender, height, weight, marital status, employment status, and education level.

### Statistical Analysis

A correlation analysis was performed to investigate the relationships between health behaviors, perceived stress, problematic internet use, and work-life balance in both groups (group 1: residents in Germany and group 2: residents in China). Moderated mediation analyses were run with health behaviors as the independent variable, work-life balance as the dependent variable, problematic internet use and perceived stress as the mediator variables, and place of residence as a moderator in both groups. Moderated mediation analyses were performed using SPSS version 24 (IBM Corp) and PROCESS [77]. Significance was accepted at an alpha level of .05.

## Results

### Preliminary Analyses

In this survey, 42.6% (374/877) of participants completed the German or English version of the questionnaire (group 1: residents in Germany), and 57.4% (503/877) of participants completed the Chinese version of the questionnaire (group 2: residents in China). When comparing health behavior items between these 2 different places of residence groups, *Physical activity* participation was higher in group 1 than in group 2 (245/374, 65.5% vs 277/503, 55.1%; *P*=.002), more participants have S*ufficient water consumption* in group 1 than in group 2 (270/374, 72.2% vs 213/503, 42.3%; *P*<.001), more participants drink alcohol on a regular basis (*alcohol consumption status*) in group 1 than in group 2 (180/374, 48.1% vs 125/503, 24.9%; *P*<.001), and there were no significant differences in *Nutrition* (*P*=.35) and *Smoking status* (*P*=.85) between the 2 groups. When comparing other variables between these 2 different places of residence groups, problematic internet use and work-life balance scores of group 2 were significantly higher than those of group 1, whereas perceived stress and health behaviors were lower in group 2. Descriptive statistics are presented in Table 1.

**Table 1:** Descriptive statistics of main study variables in the 2 groups (N=877; group 1: residents in Germany and group 2: residents in China).

Variables	Group 1, mean (SD)	Group 2, mean (SD)	Total		*t* test (df)	*P* value
			Mean (SD)	Range		
Age (years)	26.7 (12.5)	32.5 (8.4)	30.0 (10.8)	17-65	−8.24 (875)	<.001
Problematic internet use	41.8 (11.6)	44.2 (11.9)	43.2 (11.8)	20-80	−2.96 (875)	.003
Perceived stress	5.3 (1.8)	4.6 (1.5)	4.9 (1.6)	2-10	6.32 (875)	<.001
Health behaviors	3.0 (1.1)	2.9 (1.2)	2.9 (1.2)	0-5	2.44 (875)	.02
Work-life balance	16.5 (4.5)	18.0 (4.1)	17.4 (4.3)	5-25	−5.36 (875)	<.001

### Correlation Analyses in Different Groups

Table 2 displays the bivariate Pearson correlation between health behaviors, problematic internet use, perceived stress, and work-life balance. In both groups, health behaviors were positively correlated with work-life balance, whereas problematic internet use and perceived stress were negatively related to a healthy lifestyle and work-life balance.

**Table 2:** Pearson correlation of variables in 2 groups.

Group 1^a^	Group 2^b^					
	1	2	3	4	5	6
1. Gender^c^	N/A^c^	−0.07	−0.19	0.10	0.02	−0.04
2. Age	−0.11	N/A	−0.05	−0.36	−0.20	0.15
3. Health behaviors	−0.04	−0.06	N/A	−0.12	−0.27	0.11
4. Problematic internet use	0.15	−0.40	−0.16	N/A	0.32	−0.22
5. Perceived stress	−0.02	−0.23	−0.14	0.35	N/A	−0.32
6. Work-life balance	0.01	0.16	0.19	−0.27	−0.45	N/A

^a^Group 1: residents in Germany; correlations presented below the diagonal.

^b^Goup 2: residents in China; correlations presented above the diagonal.

^c^Gender was dummy coded such that 1=female and 2=male. *r*≥.15, *P*=.01; 0.10≤ *r*≤.14, *P*=.05.

^d^N/A: not applicable.

### Testing for Mediation Effects

The first aim of the study was to examine whether problematic internet use and perceived stress mediate the relationship between a healthy lifestyle and work-life balance in general. Two possible serial mediation models and one parallel model were estimated and assessed to investigate the mediator roles of problematic internet use and perceived stress.

#### Serial Mediation

**Figure 1:**
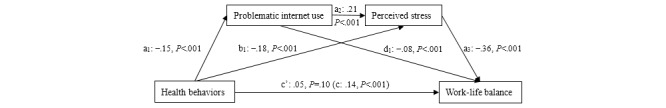
Serial mediation model and path coefficients predicting work-life balance. Path a_1_ → a_2_ → a_3_ is the full serial mediation path (1). Path a_1_d_1_ is the path from health behavior to work-life balance through problematic internet use. Path b_1_a_3_ is the path from health behavior to work-life balance through perceived stress.

Serial mediation assumes “a causal chain linking the mediators, with a specified direction of causal flow” [78]. Model 6 of PROCESS was used to test 2 possible chains: (1) health behavior → problematic internet use → perceived stress → work-life balance and (2) health behavior → perceived stress → problematic internet use → work-life balance. The proposed research model for chain 1 and results after controlling for covariates (age and gender) are presented in Figure 1. The mediation effect accounted for 19% of the total effect: *F*_5,871_=39.78; *P*<.001. The residual direct effect was not significant (c’ in Figure 1). There were significant indirect paths from health behaviors to work-life balance through problematic internet use (a_1_d_1_, β=.01, 95% CI 0.002 to 0.03) and perceived stress (b_1_a_3_, β=.06, 95% CI 0.04 to 0.09). Furthermore, the indirect path from health behaviors to work-life balance through both problematic internet use and perceived stress was also significant (a_1_a_2_a_3_, β=.01, 95% CI 0.01 to 0.02). When substituting the order of the mediators to test chain 2, the indirect path remained significant with *P*<.001, but the effect was weaker (β=.003, 95% CI 0.001 to 0.01).

#### Parallel Mediation

Parallel mediation assumes that problematic internet use and perceived stress do not influence each other when they mediate the relationship between health behaviors and work-life balance. Model 4 of PROCESS [77] was used to examine parallel mediation. The results of all participants are displayed in Table 3. After controlling for covariates (ie, age and gender), the results revealed that health behaviors were negatively associated with problematic internet use (model 2), which in turn was also interrelated with work-life balance (model 4). In addition, health behaviors were found to be negatively associated with perceived stress (model 3), which was also linked negatively to work-life balance (model 4).

The residual direct effect was not significant, with *P=*.10 (model 4). The indirect effect of problematic internet use was β=.01, 95% CI 0.002 to 0.03, and the indirect effect of perceived stress was β=.08, 95% CI 0.05 to 0.11. Most variance could be explained in model 4, predicting work-life balance with health behaviors, problematic internet use, and perceived stress.

**Figure 2:**
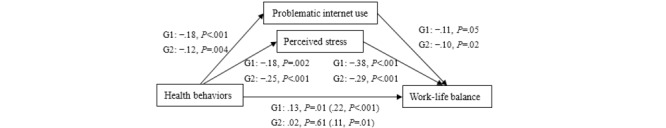
Multiple mediation model with standardized regression coefficients predicting work-life balance in both groups. G1, Group 1: residents in Germany; G2, Group 2: residents in China.

Results exploring the mediation effects in group 1 and group 2 are shown in Figure 2. In group 1, the residual direct effect was still significant (β=.13; *P*=.01) after including the mediators indicating a partial mediation only. The indirect effect of problematic internet use was β=.02, 95% CI 0.003 to 0.05; the indirect effect of perceived stress was β=.07, 95% CI 0.03 to 0.11. The mediation effect accounted for 23% of the total effect (*F*_5,368_=22.48; *P*<.001).

In group 2, the residual direct effect was not significant (β=.02; *P*=.61) when including the mediators, indicating a full mediation. The indirect effect of problematic internet use was β=.01, 95% CI 0.001 to 0.04; the indirect effect of perceived stress was β=.07, 95% CI 0.04 to 0.11. The mediation effect accounted for 12% of the total effect (*F*_5,497_=14.06; *P*<.001).

**Table 3:** Mediation effects of health behaviors on work-life balance.

Predictors	Model 1^a^ (work-life balance)		Model 2^b^ (problematic internet use)		Model 3^c^ (perceived stress)		Model 4^d^ (work-life balance)	
	β (95% CI)	*P* value	β (95% CI)	*P* value	β (95% CI)	*P* value	β (95% CI)	*P* value
Gender^e^	.04 (−0.10 to 0.18)	.53	.14 (0.02 to 0.27)	.03	−0.10 (−0.23 to 0.03)	.14	.02 (−0.11 to 0.15)	.76
Age (years)	.02 (0.01 to 0.03)	<.001	−0.03 (−0.04 to −0.03)	<.001	−0.03 (−0.03 to −0.02)	<.001	.01 (0.001 to 0.01)	.02
Health behaviors	.14 (0.08 to 0.21)	<.001	−0.15 (−0.22 to −0.09)	<.001	−0.21 (−0.28 to −0.15)	<.001	.05 (−0.01 to 0.12)	.10
Problematic internet use	N/A^f^	N/A	N/A	N/A	N/A	N/A	−0.08 (−0.15 to −0.01)	.02
Perceived stress	N/A	N/A	N/A	N/A	N/A	N/A	−0.36 (−0.42 to −0.29)	<.001

^a^*R^2^*=0.06, *F*_3,873_=17.73, *P*<.001.

^b^*R^2^*=0.14, *F*_3,873_=47.55, *P*<.001.

^c^*R^2^*=0.11, *F*_3,873_=36.83, *P*<.001.

^d^*R^2^*=0.19, *F*_5,871_=39.78, *P*<.001.

^e^Gender was dummy coded with 1=female and 2=male.

^f^N/A: not applicable.

### Testing for Mediated Moderation

The second aim of the study was to examine whether place of residence moderates the relationship between health behavior and work-life balance. This was tested with a mediated moderation analysis (model 8) of PROCESS [77], and the results are displayed in Table 4. In model 1, the total effect of the health behaviors on work-life balance was significant, and this association was not moderated by the place of residence. In model 2, the direct effect of health behaviors on problematic internet use was significant, and this association was also not moderated by the place of residence. In model 3, the direct effect of health behaviors on perceived stress was significant, and this association was also not moderated by the place of residence. In model 4, the direct effect of health behaviors on work-life balance was significant, and there was a significant moderation effect of the place of residence.

**Figure 3:**
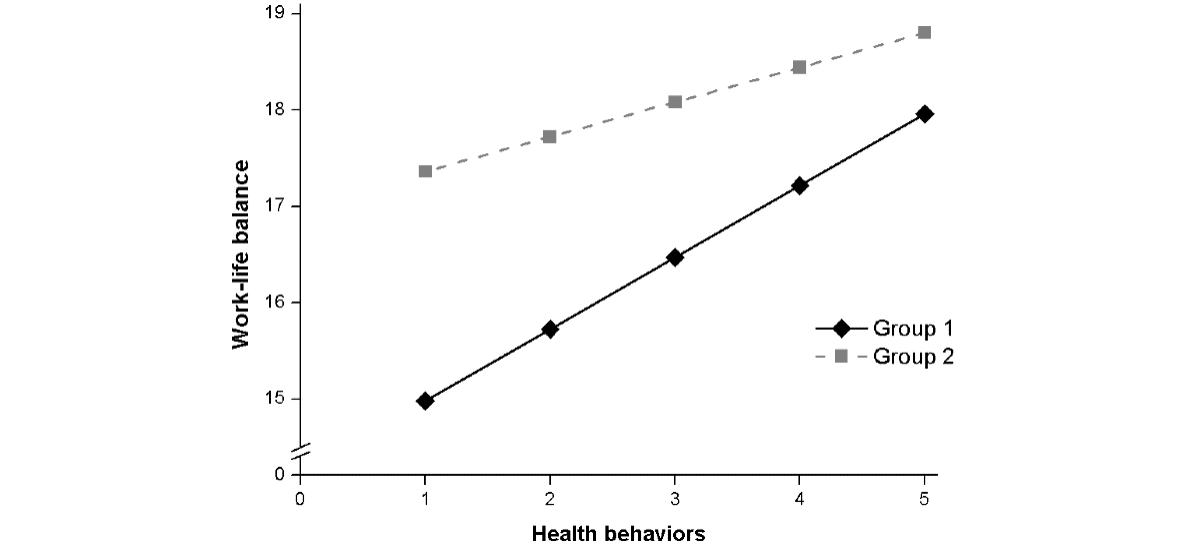
Interaction of health behaviors and residence on work-life balance (Group 1: residents in Germany; Group 2: residents in China).

To further demonstrate the pattern of this moderation effect, the interaction effect by simple slopes between health behaviors and place of residence on work-life balance is plotted in Figure 3. The simple slope test revealed that in group 1, the interrelation between health behaviors and work-life balance was significant (β=.19; *P*<.001), whereas this relation was weaker in group 2 (β=.11; *P*=.01). All predictors accounted for 20% of the variance in work-life balance in this model.

**Table 4:** Mediated moderation effects of health behaviors on work-life balance.

Predictors	Model 1^a^ (work-life balance)		Model 2^b^ (problematic internet use)		Model 3^c^ (perceived stress)		Model 4^d^ (work-life balance)	
	β (95% CI)	*P* value	β (95% CI)	*P* value	β (95% CI)	*P* value	β (95% CI)	*P* value
Gender^e^	.03 (−0.10 to 0.17)	.63	.14 (0.02 to 0.27)	.03	−0.10 (−0.23 to 0.03)	.13	.02 (−0.11 to 0.14)	.82
Age (years)	.02 (0.01 to 0.02)	<.001	−0.04 (−0.04 to 0.00)	<.001	−0.02 (−0.03 to −0.02)	<.001	.01 (−0.00 to 0.01)	.15
Health behaviors	.15 (0.09 to 0.22)	<.001	−0.15 (−0.21 to −0.08)	<.001	−0.22 (−0.28 to −0.16)	<.001	.07 (0.003 to 0.13)	.04
Residence	.15 (0.08 to 0.21)	<.001	.19 (0.13 to 0.25)	<.001	−0.16 (−0.22 to −0.10)	<.001	.11 (0.05 to 0.18)	<.001
Problematic internet use	N/A^f^	N/A	N/A	N/A	N/A	N/A	−0.10 (−0.17 to −0.04)	.003
Perceived stress	N/A	N/A	N/A	N/A	N/A	N/A	−0.34 (−0.40 to −0.27)	<.001
Health behaviors × residence	−0.05 (−0.12 to 0.01)	.11	−0.04 (−0.03 to 0.10)	.25	−0.04 (−0.10 to 0.03)	.28	−0.06 (−0.12 to 0.00)	.05

^a^*R^2^*=0.08, *F*_5,871_=15.09, *P*<.001.

^b^*R^2^*=0.18, *F*_5,871_=37.15, *P*<.001.

^c^*R^2^*=0.14, *F*_5,871_=27.97, *P*<.001.

^d^*R^2^*=0.20, *F*_7,869_=31.04, *P*<.001.

^e^Gender was dummy coded with 1=female and 2=male.

^f^N/A: not applicable.

## Discussion

### Principal Findings

An online questionnaire assessed residents from Germany (n=374) and China (n=503) in 3 languages (German, English, and Chinese). On a mean level, individuals in Germany reported less problematic internet use and more health behaviors than individuals in China; however, they also had lower work-life balance and higher perceived stress. This study is one of the first studies to explore the potential mechanisms through which health behaviors impact work-life balance [10,26,79]. Results revealed that health behaviors were indirectly related to work-life balance through perceived stress and problematic internet use, and the mediator role of problematic internet use was not prominent in either of the serial models or the parallel model. There was a partial mediation in Germany (group 1) and a full mediation in China (group 2). The relation between health behaviors and work-life balance was significant in group 1, whereas it was weaker in group 2. This study utilized the *compensatory carry-over action model* as the underlying understanding in investigating how health behaviors interrelate with employees’ work-life balance.

### Mediator Roles of Stress and Problematic Internet Use

When testing the mediator roles of stress and problematic internet use, in both serial and parallel mediation models, the residual direct effect was not significant, which indicated that problematic internet use and perceived stress fully mediated the indirect association between health behaviors and work-life balance. In the serial mediation models, the chain health behavior → problematic internet use → perceived stress → work-life balance was superior to the chain health behavior → perceived stress → problematic internet use → work-life balance. However, the effect size was small. Neither of the proposed serial models revealed a large mediating effect. In the parallel model, results exploring the mediation effects in the Germany group and the China group revealed that in the Germany group, the residual direct effect was significant. This may indicate that problematic internet use and perceived stress partially mediated the indirect association between health behaviors and work-life balance. In the China group, the residual direct effect was not significant, which is because problematic internet use and perceived stress fully mediated the indirect association between health behaviors and work-life balance.

Although health behaviors in general were better in Germany than in China, the work-life balance score was lower. According to the model, this was because of the significant difference in the mediator-perceived stress, which was much higher in Germany than in China. This is in line with previous studies [79].

The mediator role of perceived stress in this study is consistent with previous studies, which found that perceived stress could be mitigated by health behaviors [9] and affected work-life balance negatively [10]. Thus, perceived stress management is of high importance. This result also supports the *compensatory carry-over action model*, as work-life balance is not only associated with predictor variables related to health behaviors but also with mediator variables, such as perceived stress. To the best of our knowledge, this is the first study that clarifies the mediator roles of perceived stress and problematic internet use in a healthy lifestyle consisting of different single health behaviors and work-life balance in different countries of residence.

Although previous studies have found health behaviors to be negatively associated with problematic internet use [7-9], which in turn affects work-life balance [80-82], in this study, the meditation role of problematic internet use was not prominent compared with the perceived stress mediator. This may be because of the interaction between perceived stress and problematic internet use. This study revealed that there was a significant relationship between problematic internet use and perceived stress. Previous studies have indicated that problematic internet use has a direct impact on perceived stress [40,41]; however, perceived stress may also come from other aspects, such as occupational, interpersonal, or financial stresses [83]. Moreover, previous research has demonstrated that perceived stress plays an important role in problematic internet use [44,45]. It is difficult to judge which factor affects the other, as it depends on circumstances, and this study was only cross-sectional.

Apart from perceived stress and problematic internet use, the path between health behaviors and work-life balance may also be explained by other variables, such as gender. Gender differences have been found in relation to health behaviors in previous studies [84], and they have also been highlighted in work-life balance studies [5]. Some studies have found that women often have more difficulty maintaining a balance because of pressures at work and social role demands at home [85,86]. However, other studies have indicated that men and women report similar levels of work-life balance [87]. Other studies have shown that women tend to present a more positive work-life balance attitude compared with men [88]. Further studies that focus on the organization of work taking the employee’s gender difference into account are recommended.

### Moderation Role of Place of Residence

This study found that problematic internet use score in China was higher than that in Germany. This is in line with the previous studies, which found that people of an Asian background, such as Chinese, have higher problematic internet use scores than people from Europe or the United States [66,89].

Countries of residence rather than countries of birth were compared in this study, as it has been found that the effects on people’s individual life from the place of residence appear to be higher than those from the place of birth [90]. From a closer look at country differences in health behaviors, it seems that the country where one lives provides the context within which a certain kind of behavior is shaped. In the case of Germany, it would be of interest to study the role of the country as a context variable in the health life and behavior of its residents, regardless of their cultural origin. Not only health behaviors but also work-life balance was different because of the country of residence, which involves influences of the natural environment, language, religion, custom, and particular ways of life [91,92]. This study found that the indirect association between health behaviors and work-life balance was moderated by the place of residence. The relation between health behaviors and work-life balance was significant in residents living in Germany, whereas it was weaker in residents in China.

This finding is in line with and further supports previous studies that explored health behaviors related to work-life balance [12,14,17,26,93]. This study also replicates studies that examined work-life balance across different societies [53]. The result showing that the relation between health behaviors and work-life balance was significant in Germany, whereas it was weaker in China, supports the *compensatory carry-over action model*. The model permits different applicability in 2 distinct countries of residence, from which, besides health behaviors, work-life balance could be affected by different factors. Previous studies have found that in Eastern countries, gender socialization plays an important role in one’s perception toward work-life balance [53]. Moreover, although age was controlled in this study, other variables such as family responsibility status may be associated with work-life balance as well. Previous studies have indicated that older and married individuals felt less stressed than younger and unmarried people [75]. The possibility of married status in many Chinese participants might modulate the effect of health behaviors on work-life balance as well. This argument should be further explored in future research.

In this study, compared with residents in China, more people in Germany engaged in physical activity for at least 2.5 hours per week and drank enough water, whereas more people in Germany drank alcohol than people in China. These findings are in line with previous studies that have investigated health behaviors in Western countries and Eastern countries [63]. The moderation of the place of residence provides a broad perspective toward the promotion of work-life balance among 2 very diverse groups of countries of residence. By contrasting residents from China and Germany as representatives of prevailing Eastern and Western lifestyles, respectively, we may be able to gain a more global perspective in this issue. Professionals in the medical field should advise their patients in China to engage in more health behaviors and patients in Germany to find ways of lowering perceived stress to balance their lifestyle more adequately.

### Limitations

Some limitations of this study should be addressed. First, this was a cross-sectional survey, which limits causal inferences. Moreover, there was a significant age difference between participants in the 2 groups. Although age and gender were included as control variables in this study, other variables such as family responsibility status may be associated with different ages and may impact the results as well. Second, health behaviors only included physical activity, nutrition and water intake, and smoking and alcohol status. However, other behaviors, such as dental hygiene adherence and sexual behaviors, are also related to health and may have an influence. Third, perceived stress was measured with only two items by using the shortened version of the measurement scale, and the Cronbach alpha was not high. The accuracy may not be as good as a full version. Finally, the data were mainly collected within Germany and China. Although participants with a migrant background were able to complete the English version of the questionnaire, the sample range was still limited. Moreover, the years of residence were not controlled, but it may also impact individuals’ behaviors. Further studies should overcome these limitations by using longitudinal and experimental designs and considering more aspects and potential factors. It would also be valuable to include more people with different backgrounds regarding their country of residence while controlling other influences.

### Conclusions

In summary, this study sheds light on how people’s health behaviors interrelate with work-life balance, perceived stress, and problematic internet use. It provides evidence that health behaviors are indirectly related to work-life balance through perceived stress and problematic internet use. This study revealed a partial mediation among residents in Germany and a full mediation among residents in China. The relation between health behaviors and work-life balance was stronger in Germany, whereas it was weaker in China. Besides performing health behaviors, helping people to relieve their perceived stress and spend only healthy amounts of time with the internet seems essential to maintain a better work-life balance. Future research should be performed in different countries and in different languages, and should be well grounded on a culture theory, such as Hofstede’s cultural dimensions theory [48,49].

## Abbreviations

ISS-10r: Internet Addiction Questionnaire
